# IL-7 in colostrum is associated with atopic dermatitis at 9 months of age: A birth cohort in Japan

**DOI:** 10.5415/apallergy.0000000000000219

**Published:** 2025-08-13

**Authors:** Saori Abe, Daisuke Furushima, Kazutoshi Sayama, Naoki Shimojo

**Affiliations:** 1 Division of Technology, Shizuoka University, Shizuoka, Japan; 2 Department of Applied Life Sciences, Faculty of Agriculture, Graduate School of Agriculture, Graduate School of Science and Technology, Shizuoka University, Shizuoka, Japan; 3 Department of Community Health Nursing, Faculty of Medicine, School of Health Science, Kagoshima University, Kagoshima, Japan; 4 Department of Drug Evaluation and Informatics, Graduate School of Pharmaceutical Sciences, University of Shizuoka, Shizuoka, Japan; 5 Department of Pediatrics, Graduate School of Medicine, Chiba University Hospital Allergy Center, Chiba, Japan

**Keywords:** Atopic dermatitis, birth cohort, breast milk, IL-7, infancy

## Abstract

**Background:**

CCL25, CCL28, and IL-7 are present in breast milk and are known to contribute to the infant’s thymus function and mucosal immune system. However, little is known about the relationship between the concentration of these cytokines/chemokines in breast milk and the development of atopic dermatitis (AD) in infancy.

**Objective:**

The purpose of this study was to investigate whether the concentration of these cytokines/chemokines in breast milk is related to the development of AD in infants.

**Methods:**

In this study, we measured the concentrations of cytokines/chemokines in colostrum collected within 5 days of birth and in breast milk collected at 1-month of birth in 281 infants belonging to a birth cohort using sandwich ELISA, and analyzed the relationship with the development of AD in infants at 9 months of age.

**Results:**

There was no association between CCL25 and CCL28 levels in breast milk and 9-month AD. On the other hand, IL-7 levels in colostrum ingested by AD infants were significantly higher than those ingested by non-AD infants (median, ng/mL: 0.186 vs 0.119, Mann-Whitney *U* test, *P* < 0.01). In addition, IL-7 showed a significant dose-dependent relationship with AD. This relationship was only observed in mothers with allergies.

**Conclusion:**

The results of this study show that IL-7 levels in breast milk may offer insights into the pathophysiological mechanisms of infantile AD.

## 1. Introduction

Recent surveys in Japan have reported that the prevalence of atopic dermatitis (AD) is 15.6% across all age groups and 13.5% in children under 4 years of age [1]. AD in infancy is the starting point of the allergic march, and understanding its pathophysiology is important for the prevention of allergic diseases, including those in adults. Several environmental factors are associated with the onset of AD in infancy, including breastfeeding. However, the association between infant nutrition and AD remains unclear [2]. The heterogeneity of research results is thought to be related to the quality and quantity of immune-active substances in breast milk.

Our previous study first identified the presence of CCL25 in human breast milk [3]. In addition to CCL25, CCL28, and IL-7 in breast milk have been implicated in neonatal thymic maturation/mucosal immune system [4–7]. Cytokine/chemokine levels in breast milk vary according to the type of cytokine/chemokine, duration of lactation, and the individual woman. Although their levels are relatively low, TGF-β, IL-6, and IL-10, which are involved in the neonatal immune system, have been reported to be associated with allergic outcomes in early childhood [8]. Therefore, CCL25, CCL28, and IL-7 levels in breast milk may also influence allergic outcomes in breastfed infants. However, no studies have evaluated the relationship between CCL25 and CCL28 in breast milk and allergic disease in infants. Furthermore, only 1 study has examined the association between IL-7 in breast milk and the development of AD in infants [9]. The aim of this study was to investigate whether CCL25, CCL28, and IL-7 in breast milk are associated with AD at 9 months of age.

## 2. Materials and methods

### 2.1. Birth cohort

A birth cohort was designed to investigate whether skin care and synbiotics use in infants prevent AD at 9 months of age (Katsushika Study, 2 × 2 factorial randomized nontreatment controlled trial study) [10]. The study recruited 549 pairs of mothers and children who had completed a full-term pregnancy and delivered by vaginal delivery or cesarean section at Katsushika Red Cross Maternity Hospital Tokyo (Japan) between October 2012 and March 2014. Mothers and infants were recruited from the general population for this study. In this study, no intervention was associated with the prevention of the development of AD. In response to this finding, the present study analyzed the association with the development of AD at 9 months of age using milk samples from 281 pairs of mothers and infants who were exclusively or almost exclusively breastfed for the first 4 months of life. “Almost exclusively breastfed” refers to infants who are given only a few feedings of formula until 4 months of age. Information on maternal characteristics was collected from questionnaires administered during hospitalization.

### 2.2. Ethical considerations

Ethical approval for this study was provided by the Chiba University Ethics Committee (reference no. 2067), 1-8-1, Inohana, Chuo-ku, Chiba-shi, Chiba, 260-0856, Japan (Chairperson Dr. Toshinori Nakayama) on 29 March 2017 and the Shizuoka University Ethics Committee (reference no. 17-44), 836 Ohya, Suruga-ku, Shizuoka, 422-8529, Japan (Chairperson Prof Toshihiko Dozono) on 15 February 2018.

### 2.3. Sampling and assay for breast milk cytokines/chemokines

Breast milk was collected within 5 days of birth (colostrum) and at 1-month of age (1-month milk). All specimens were stored at −80°C within 12 hour after collection. Immediately before the determination of cytokines/chemokines in breast milk, specimens were thawed and centrifuged at 15,000 rpm for 5 minute, after which only whey was separated. CCL25, CCL28, and IL-7 concentrations in whey were measured with commercially available ELISA kits (The Human CCL25 DuoSet ELISA kit R&D, The Human CCL28 DuoSet ELISA kit R&D, The Human IL -7 DuoSet ELISA kit R&D) according to the manufacturer’s manual. Each assay was performed using high-affinity ELISA plates (CostarAssay Plate, 96 well, Corning, USA). The minimum detection limits for CCL25, CCL28, and IL-7 in breast milk were defined as the lowest value of the calibration curve, respectively. Samples in which no cytokines were detected were set at 0 ng/mL when analyzed; the percentages of colostrum in which CCL25, CCL28, and IL-7 were detected were 0%, 80.9%, and 89.3%, respectively, and in 1-month milk were 0%, 75.3%, and 69.0%, respectively.

### 2.4. Diagnosis of AD

AD in 9-month-old infants was diagnosed by a pediatrician according to the guidelines of the Japanese Dermatological Association.

### 2.5. Statistical analysis

Continuous variables were expressed as mean (SD) or median (IQR), as appropriate. Categorical variables were presented as numbers and percentages. Continuous variables were compared by Student’s *t* test or Mann-Whitney *U* test, and categorical variables were compared by Pearson’s chi-square test. To examine the association between cytokine/chemokine levels in breast milk and the incidence of AD in infants, cytokine/chemokine levels were divided into 4 groups (Q1–Q4) according to quartiles, and the incidence of AD between groups was compared by the Cochran-Armitage trend test, with *P* value < 0.05 being considered significant. All statistical analyses were performed using IBM SPSS Statistics ver. 29.

## 3. Results

### 3.1 Possible factors related with development of AD in infants

Data from 276 children whose colostrum/1-month breast milk were collected and who were diagnosed with or without AD at 9 months were used in the analysis. Possible factors associated with the development of AD in infants are summarized in Table 1: maternal age, maternal history of allergies, cesarean section, gestational age, history of atopy of family members, timing of complementary feeding introduction, and randomized controlled trial intervention allocations in our previous study [10]. In particular, a maternal history of allergies was significantly associated with AD in 9-month-old infants (Pearson’s chi-square test, *P* = 0.016). On the other hand, any other factors were not significantly associated with the onset of AD (Table 1).

**Table 1.** Participants characteristics in nonatopic dermatitis and atopic dermatitis groups at 9 months of age

Characteristics	Non-AD group	AD group	*P* value
	n = 236	n = 40	
Maternal age Mean of year (SD)	34 (4.23)	34 (4.22)	0.976
Maternal history of allergies	98 (41.5)	25 (62.5)	0.016
Yes, n (%)			
Cesarean section	45 (19.1)	11 (27.5)	0.220
Yes, n (%)			
Gestational age	39.1 (1.16)	39.0 (1.34)	0.465
Mean of month (SD)			
Timing of complementary feeding introduction	5.63 (0.724)	5.40 (0.709)	0.060
Mean of month of age (SD)			
History of atopy of family members	3 (46.4)	8 (80)	0.080
Yes, n (%)			
Synbiotics and skincare^*^	58 (24.6)	10 (25.0)	0.821
Yes, n, (%)			
Synbiotics^*^	50 (21.1)	11 (27.5)	0.821
Yes, n, (%)			
Skincare^*^	66 (28.0)	10 (25.0)	0.821
Yes, n, (%)			
Nonintervention^*^	62 (26.3)	9 (22.5)	0.821
Yes, n, (%)			

Maternal history of allergies in the table was represented mothers with a history of 1 or more of AD, asthma, or allergic rhinitis. Means between groups were compared by Student’s *t* test, and the proportions between groups were compared by Pearson’s chi-squared test/Fisher’s exact test.

AD, atopic dermatitis; RCT, randomized controlled trial.

* Synbiotics and skincare, synbiotics, skincare, and nonintervention were represented in RCT intervention allocations in our previous study.

### 3.2 Cytokine/chemokines levels in colostrum and 1-month milk

**Figure 1.**
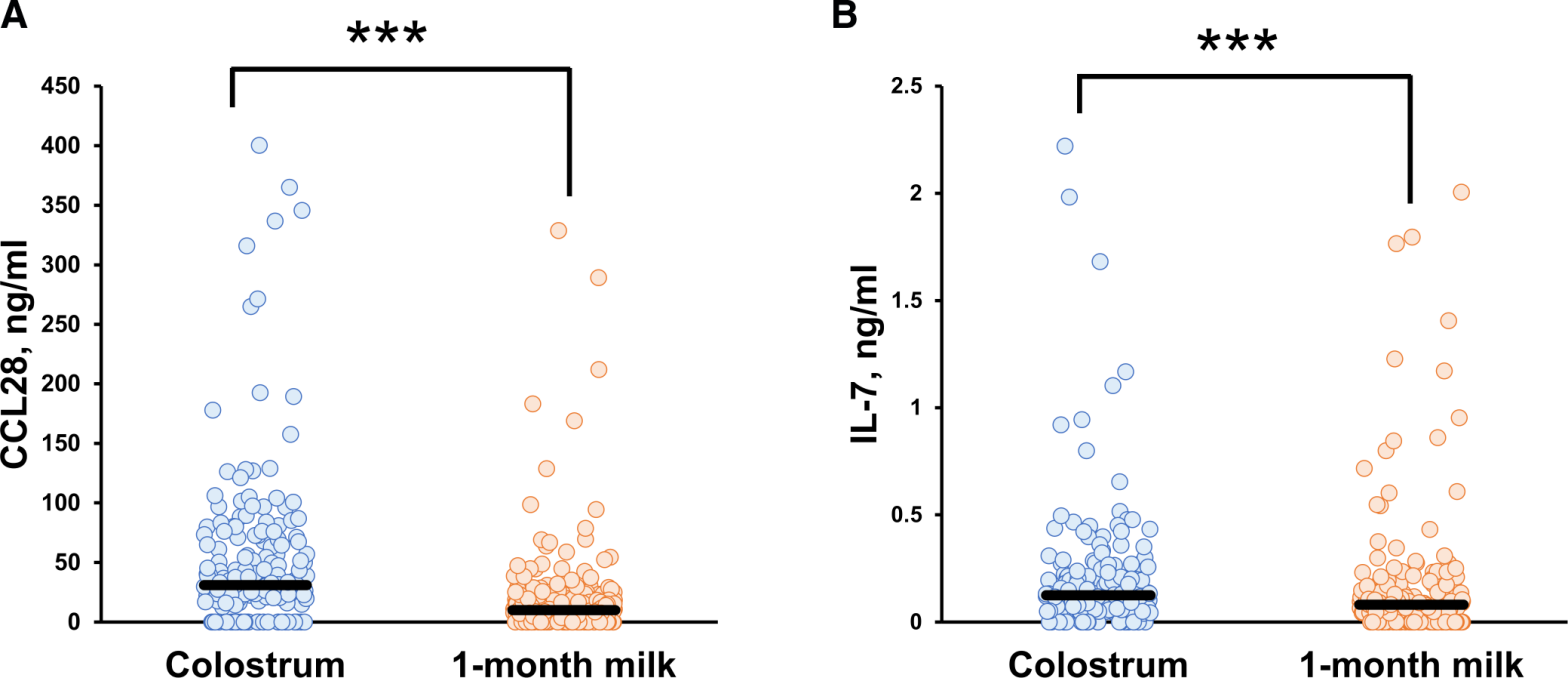
Cytokine/chemokine levels in colostrum and 1-month breast milk are shown: (A) CCL28, (B) IL-7. Median values of cytokines/chemokines are shown as bars; 3.545, 3.562 ng/mL above the upper limit in (B) are present in 1-month breast milk. ***showed significant differences at *P* < 0.001. Median CCL28/IL-7 values were compared between colostrum and 1-month milk (Mann-Whitney *U* test, *P* < 0.001).

Figure 1 shows the cytokine/chemokine concentrations in colostrum and 1-month breast milk. Only CCL28 and IL-7 were detected in colostrum, and their concentrations were significantly higher than those in 1-month breast milk (Fig. 1A, B) (Mann-Whitney *U* test, *P* < 0.001). The median (IQR) of CCL28 levels was 31.0 (20.9–62.5) ng/mL in colostrum and 10.0 (4.70–21.3) ng/mL in 1-month milk, and the median (IQR) of IL-7 levels was 0.13 (0.063–0.222) ng/mL in colostrum and 0.08 (0.00–0.158) ng/mL in 1-month milk.

### 3.3 Cytokine/chemokine levels in breast milk of non-AD and AD infants

**Figure 2.**
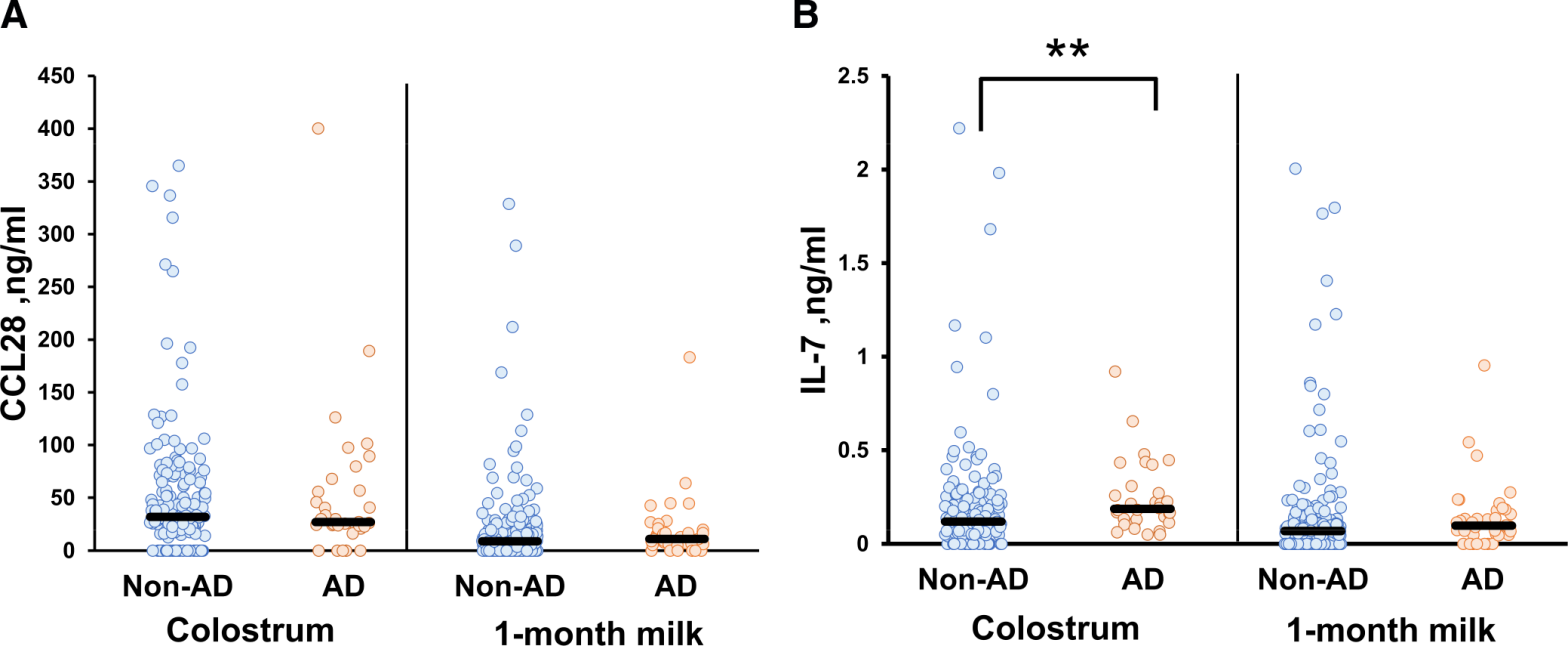
Cytokine/chemokine levels in breast milk ingested by AD or non-AD children. Cytokine/chemokine levels in colostrum and 1-month breast milk of AD and non-AD children are shown: (A) CCL28, (B) IL-7. Median cytokine/chemokine values are indicated by bars. 3.545 and 3.562 ng/mL above the upper limit in (B) were found in 1-month breast milk of non-AD infants. **showed a significant difference at *P* < 0.01; colostral IL-7 levels in the AD group were significantly higher than those in the non-AD group (Mann-Whitney *U* test, *P* < 0.01). AD, atopic dermatitis.

Figure 2 compares CCL28 and IL-7 levels in breast milk ingested by AD and non-AD infants; CCL28 levels in colostrum/1-month milk were similar between non-AD and AD children (Mann-Whitney *U* test, *P* > 0.05) (Fig. 2A). In contrast, IL-7 levels in colostrum ingested by AD infants were significantly higher than those ingested by non-AD infants (median, ng/mL: 0.186 vs 0.119, Mann-Whitney *U* test, *P* < 0.01) (Fig. 2B). IL-7 levels in 1-month milk ingested by AD infants also tended to be higher than those ingested by non-AD infants (median, ng/mL: 0.097 vs 0.068, Mann-Whitney *U* test, *P* = 0.069) (Fig. 2B).

### 3.4 Percentage of breast milk with detectable cytokine/chemokine levels ingested by non-AD infants and AD infants

As shown in Table 2, the percentage of colostrum ingested by AD children with detectable IL-7 was significantly higher than that ingested by non-AD children (Pearson’s chi-square test, *P* = 0.023).

**Table 2.** The number/proportion of breast milk with a positive level of cytokine/chemokine

Cytokines		Non-AD group	AD group	*P* value
Colostrum				
CCL28	Sample, n	179	33	0.553
	Positive, n (%)	144 (80.4)	28 (84.8)	
IL-7	Sample, n	170	32	0.023
	Positive, n (%)	150 (88.2)	32 (100)	
1-month milk				
CCL28	Sample, n	216	39	0.158
	Positive, n (%)	160 (74.1)	33 (84.6)	
IL-7	Sample, n	209	40	0.115
	Positive, n (%)	141 (67.5)	32 (80.0)	

Samples in the table indicated the number of breast milk used for the assay of cytokine/chemokine. The number and percentage of positive in the table indicated samples with positive levels of cytokine/chemokine. The proportion of positive results between groups was compared by Pearson’s Chi-squared test.

### 3.5 Relationship between colostral IL-7 levels and AD in 9 months-old infants

**Figure 3.**
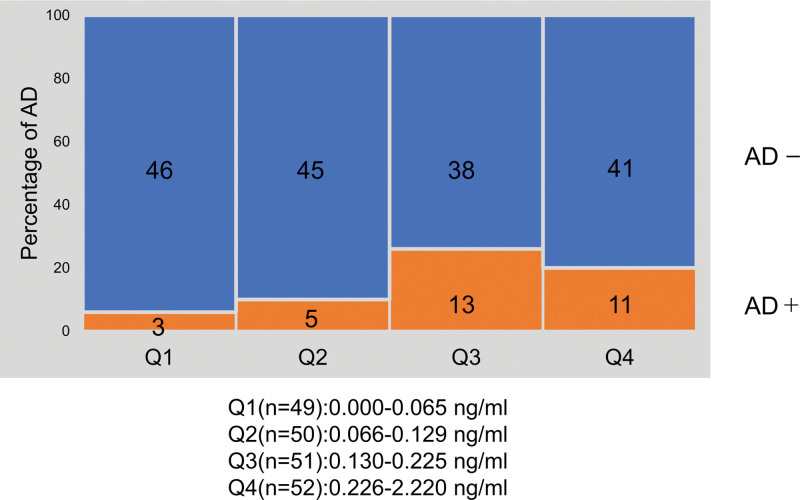
Association between IL-7 levels in colostrum and rate of AD in infants. The IL-7 concentration in breast milk was divided into 4 groups according to quartiles, and the AD rate at 9 months of age was compared among the groups. The numbers in the figure indicate the number of AD/non-AD infants in each group. The rate of AD at 9 months of age increased significantly with increasing IL-7 concentration in colostrum (Cochran-Armitage trend test, *P* = 0.039). AD, atopic dermatitis.

Figure 3 compares AD incidence rates across quartiles of IL-7 levels to examine in detail the association between the dose of colostral IL-7 and AD incidence. The results showed that colostral IL-7 was significantly associated with increased incidence of AD in infants in a dose-dependent manner (Cochran-Armitage trend test, *P* = 0.039).

### 3.6 Effect of maternal allergy on the relationship between the development of AD in 9 month-olds and IL-7 levels in colostrum

**Figure 4.**
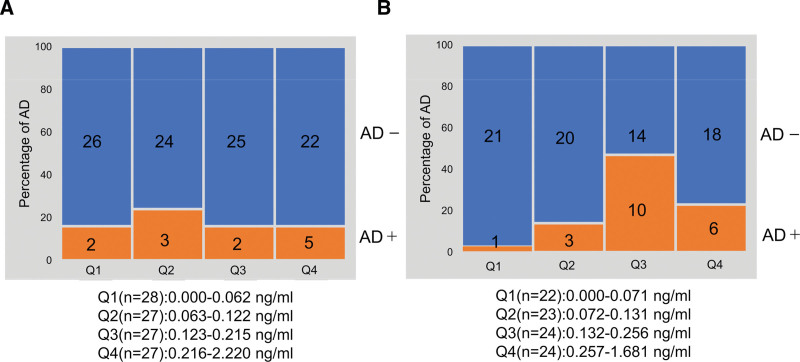
Association between IL-7 levels in colostrum and incidence of AD at 9 months of age (stratified by maternal history of allergy). (A) Incidence rate of AD in infants breastfed by nonallergic mothers (B) incidence rate of AD in infants breastfed by allergic mothers. The numbers in the figure indicated the number of AD/non-AD infants in each group. Incidence rate of AD in 9-month-old infants breastfed by allergic mothers was significantly increased with increasing colostral IL-7 levels (Cochran-Armitage trend test, *P* = 0.031). AD, atopic dermatitis.

Participants were stratified based on maternal history of allergy, and the association between colostrum IL-7 dose and AD outcome at 9 months of age was reanalyzed (Fig. 4). An association was found for allergic mothers (Cochran-Armitage trend test, *P* = 0.031) (Fig. 4B), but not found for nonallergic mothers (Cochran-Armitage trend test, *P* = 0.550) (Fig. 4A).

## 4. Discussion

In this study, CCL28 and IL-7 were detected in breast milk, but not CCL25. Only IL-7 in colostrum was associated with AD at 9 months of age, and its level was also associated with the incidence rate of AD in a dose-dependent manner. To our knowledge, only 1 study has assessed the association between IL-7 levels in breast milk and infant AD [9]. They used a multiplex assay and showed that IL-7 levels in colostrum and 1-month milk ingested by AD infants tended to be higher than those ingested by non-AD infants (colostral median, pg/mL: 27.4 vs 20.2, 1-month median, pg/mL 20.2 vs 17.2). We used a sandwich ELISA method to detect more accurate cytokine levels in breast milk. Compared to previous studies using the multiplex method, higher concentrations of colostral IL-7 were detected in the present study, showing a significant difference between the AD and non-AD groups (median, ng/mL: 0.18 vs 0.11, *P* < 0.01). Previous experimental and clinical studies have shown that IL-7 levels in breast milk are positively correlated with increased thymus size/thymocyte production in infants [11]. Recent studies have shown that there is a positive correlation between thymus size and severity of AD (SCORAD) in infants, and that SCORAD is also positively correlated with the number of lymphocytes in the patient’s blood [12]. In addition, studies in neonatal mice have shown that oral intake of IL-7 is delivered to the thymus via the gastrointestinal tract [6]. Although we did not measure thymus size, it is possible that orally ingested IL-7 affects thymus maturation and increased AD incidence in infants. In the present study, breast milk IL-7 levels were associated with the development of infant AD only in those born to allergic mothers. However, there were no significant differences in breast milk IL-7 concentration between allergic and nonallergic mothers (data not shown). This result suggested that the association between IL-7 levels in colostrum and the onset of AD was not simply a reflection of the mother’s allergic status but occurred independently of the mother’s allergic status. Other maternal factors may be involved in IL-7 levels.

So far, no studies have examined the association between CCL28 concentrations in breast milk and AD in infants. Previous studies have shown that CCL28 in breast milk increases the production of IgA into breast milk as well as TGF-β, IL-6, and IL-10 [4, 13]. IgA levels in breast milk were negatively associated with AD positivity and cow’s milk allergy [14, 15]. Thus, we expected that CCL28 in breast milk might prevent AD in infants, but no significant association between the breast milk CCL28 levels and AD was demonstrated.

In the present study, we could not detect CCL25 in breast milk, while CCL25 was detected in breast milk in our previous study [3]. One reason for this discrepancy may be the difference in storage period of breast milk, which may affect cytokine levels. In the present study, CCL28 levels were also reduced and were approximately 16.0%–50.9% of the levels detected in previous studies. In particular, the decrease in CCL28 levels was more pronounced in 1-month milk. In our previous study, the median CCL28 levels in breast milk were colostrum: 60.9 ng/mL and 1-month breast milk: 62.5 ng/mL [3], whereas in the present study, the levels were 31.0 ng/mL in colostrum and 10.0 ng/mL in 1-month milk. In addition, IL-7 levels were also reduced and were approximately 66.7%–86.7% of the levels detected in previous studies [3]. In our previous study, the median IL-7 levels were colostrum: 0.15 ng/mL and 1-month milk: 0.12 ng/mL [3], whereas in the present study, the median IL-7 levels were 0.13 ng/mL for colostrum and 0.08 ng/mL for 1-month milk. However, the degree of reduction in those concentrations was relatively smaller than CCL25 and CCL28. Walter et al. suggested that the concentration of IL-7 was less affected by the storage period of breast milk, which was consistent with our results [16]. The stability of cytokines in breast milk depends on the substance, and several studies have shown that cryopreservation changes the detected concentration [17, 18]. As CCL25 was not detected and the CCL28 level was lower in this study, the concentrations of these chemokines reduced by long-term cryopreservation. In particular, CCL25 has a very low content in breast milk, further studies by use of fresh breast milk samples are needed to investigate the association between CCL25 and AD.

The present study has several limitations. The present study was not able to investigate the diet and lifestyle habits of the mothers during pregnancy and lactation. Therefore, further studies are needed to confirm their influence on breast milk cytokines/chemokines. Nevertheless, there is no evidence so far for an association between CCL28 and IL-7 in breast milk and the development of AD in infants [9]. The present findings may be helpful in understanding the pathogenesis of AD in early childhood.

In conclusion, IL-7 levels in breast milk may offer insights into the pathophysiological mechanisms of infantile AD. Further investigation is needed to identify factors that may influence IL-7 levels in breast milk.

## Conflicts of interest

KS received a financial donation from Mr. Isamu Taguchi and Miroku CO., LTD. Note that they are not involved in decisions regarding research design, data collection, analysis and interpretation, report writing, and article submission. The remaining authors declare no conflicts of interest.

## Author contributions

Saori Abe analysed breast milk, performed statistical analysis, and wrote the article. Daisuke Furushima advised on the statistical analysis. Kazutoshi Sayama designed this study and prepared the equipment for the experiment. Naoki Shimojo provided breast milk samples and advised on the conduct of this study, statistical analysis, and writing the article.

## Acknowledgements

We would like to grateful to the mothers and their children that participated in this study. We also would like to thank the medical staff of the Katsushika Red Cross Maternity Hospital, Tokyo, for their assistance. In addition, we are grateful to Dr. Taiji Nakano, Dr. Fumiya Yamaide, Dr. Eishika Dissanayake, Mrs. Megumi Omori, and the staffs of the Department of Pediatrics, Graduate School of Medicine/Center for Preventive Medical Science, Chiba University for their assistance.
